# Topographic organization of the human and non-human primate subthalamic nucleus

**DOI:** 10.1007/s00429-015-1047-2

**Published:** 2015-04-29

**Authors:** Anneke Alkemade, Alfons Schnitzler, Birte U. Forstmann

**Affiliations:** Cognitive Science Center Amsterdam, University of Amsterdam, Nieuwe Achtergracht 129, 1018 WS Amsterdam, The Netherlands; Department of Neurology, Medical Faculty, Center for Movement Disorders and Neuromodulation, Heinrich-Heine University, Düsseldorf, Germany; Medical Faculty, Institute of Clinical Neuroscience and Medical Psychology, Heinrich-Heine University, Düsseldorf, Germany

**Keywords:** Basal ganglia, Parkinson’s disease, Deep brain stimulation, Decision making

## Abstract

Deep brain stimulation (DBS) of the subthalamic nucleus (STN) is used to relieve motor symptoms of Parkinson’s disease. A tripartite system of STN subdivisions serving motoric, associative, and limbic functions was proposed, mainly based on tracing studies, which are limited by low numbers of observations. The evidence is compelling and raises the question as to what extent these functional zones are anatomically segregated. The majority of studies indicate that there is anatomical overlap between STN functional zones. Using ultrahigh-resolution magnetic resonance imaging techniques it is now possible to visualize the STN with high spatial resolution, and it is feasible that in the near future stereotactic guided placement of electrical stimulators aided by high-resolution imaging will allow for more specific stimulation of the STN. The neuroanatomical and functional makeup of these subdivisions and their level of overlap would benefit from clarification before serving as surgical targets. We discuss histological and imaging studies, as well as clinical observations and electrophysiological recordings in DBS patients. These studies provide evidence for a topographical organization within the STN, although it remains unclear to what extent functionally and anatomically distinct subdivisions overlap.

## Introduction

**Fig. 1:**
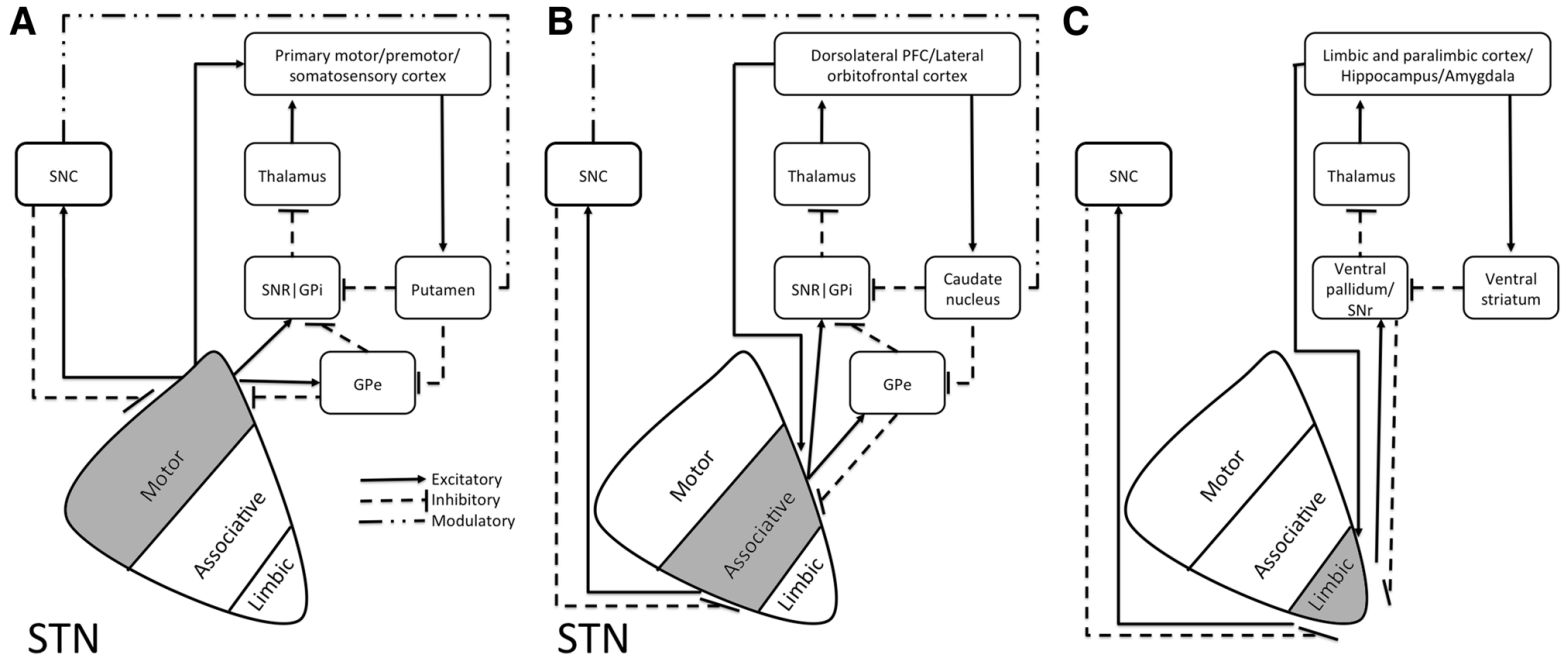
Schematic illustration of positioning of the STN and the connectivity of functional putative subdivisions within the basal ganglia thalamocortical **a** motor, **b** associative, and **c** limbic circuits. Adapted from (Temel et al. 2005). Based on tracing studies in monkeys using both antero- and retrograde tracings, anatomofunctional subdivisions of the STN have been proposed. Cortical areas involved in the motor circuitry include the primary motor, premotor, and somatosensory cortex (**a**). Cortical areas involved in the associative circuitry include the dorsolateral prefrontal cortex, as well as the lateral orbitofrontal cortex (**b**). The associative circuits include the direct and indirect pathway. The direct pathway runs via the internal segment of the Globus Pallidus (GPi) and reticular part of the Substantia Nigra (SNr) to the ventroanterior (VA) and centromedian (CM) nuclei of the thalamus, and the circuit is closed by the thalamocortical pathway back to the dorsolateral prefrontal cortex (DLPC) and the lateral orbitofrontal circuit back to the lateral orbitofrontal cortex (LOFC). The indirect pathway encompasses a projection from the external part of the globus pallidus (GPe) to the STN and GPi/SNr. The limbic circuitry involves limbic and paralimbic cortices as well as hippocampus and amygdala (**c**) (Temel et al. 2005). Although subdivisions are anatomically separated in this illustration, evidence from tracing studies point towards significant overlap of these subdivisions (Haynes and Haber 2013)

The subthalamic nucleus (STN) lies deep within the brain on top of the cerebral peduncle and plays a central role in both the direct and indirect pathway of the basal ganglia (Temel et al. 2005) (Fig. 1). Even though the nucleus itself is not degenerated in Parkinson’s disease, it plays an important role as a target for deep brain stimulation (DBS) in its treatment. Targeting of the STN to modulate its function using DBS provides relief of severe motor symptoms and significantly improves quality of life in Parkinson’s patients (Limousin et al. 1995; Volkmann et al. 2001; Rodriguez-Oroz et al. 2004). The exact mechanism through which DBS exerts its clinical effect is still under debate (Temel et al. 2007). DBS in the STN is not without risk. Despite differences in definitions between individual research groups, the occurrence of cognitive/psychiatric side is consistently reported (Benabid et al. 2009). Case studies describe rare neuropsychiatric side effects including apathy, compulsive behavior, hypersexuality, cognitive dysfunction as well as clinical depression including suicide (Temel et al. 2005), and a recent, larger study reports emotional lability in patients receiving DBS in the STN as well as in the internal segment of the globus pallidus (GPi) (Odekerken et al. 2013).

We would like to note that a direct association between DBS surgery and an increased risk for suicide ideation and behavior has not been shown (Weintraub et al. 2013). Interestingly, the side effects correspond to other functions of the basal ganglia and the STN in addition to its’ role in motor control (Temel et al. 2005).

The STN is considered the main relay station of the indirect pathway of the basal ganglia. Its action together with signals from the direct inhibitory GABA-ergic pathway modulates the activity of GPi and the reticular part of the substantia nigra (SNr) (DeLong and Wichmann 2007). The STN in turn is under the control of the GPe, cerebral cortex, compact part of the substantia nigra (SNpc), dorsal raphe nucleus, pedunculopontine tegmental nucleus, and the centromedian/parafascicular thalamic complex (Fig. 1, Parent and Hazrati 1995a, b).

A number of studies have investigated the anatomofunctional organization within the STN. The tripartite subdivision hypothesis proposes the existence of subdivisions within the STN, corresponding to limbic, associative and motor functions. We have recently raised the question whether the tripartite subdivision hypothesis of the STN needs revision, which has already led to a response from other research groups underlining the interest in the discussion within the research field (Alkemade and Forstmann 2014; Lambert et al. 2015). The aim of this review is to critically re-evaluate available literature on the neuroanatomical organization within the STN in an attempt to determine to what extent anatomofunctional subdivisions within the STN show overlap, and whether they support the existence of (largely) separated subdivisions. We will summarize findings from tracing studies, histological studies in human and non-human primates, and from in vivo imaging studies in healthy participants and pathophysiological observations.

We would like to note that rodent studies have been invaluable in increasing understanding of the STN. Animal models that faithfully recapitulate the pathological hallmarks of Parkinson’s disease are crucial for the understanding of the pathogenic pathways in vivo (Lee et al. 2012). However, as is the case for most if not all rodent models for neuropsychiatric and neurodegenerative disorders, no genetic mouse model recapitulates all features of human Parkinson’s disease (Lee et al. 2012). In addition, the STN’s anatomy shows clear species differences. In rats, the STN is considered an open nucleus which means that its’ dendrites extend into brain areas outside the STN (Afsharpour 1985). In primates, STN dendrites are almost exclusively restricted to the nucleus itself, and the STN is therefore considered to be closed (Rafols and Fox 1976). We acknowledge important progress made in the field by rodent studies, but in the present review we will focus on data obtained in primates. We made this choice, since although to a large extent human and rodent data are comparable, there may be small anatomical differences present between subdivisions in distinct species. These small differences may have large consequences for the efforts that are made to specifically target the dorsolateral part of the STN in order to minimize side effects associated with the unwanted stimulation of associate and limbic parts of the STN.

Data have been obtained in humans and non-human primates using a number of research approaches. Each approach has its merits, as well as limitations, which is important for the interpretation of the research findings (Table 1). We have structured the evidence for a topographical organization within the STN according to the applied research approach.

**Table 1:** Strengths and weaknesses of research techniques

Research technique	Strengths	Weaknesses
Tracing	High level of anatomical detail, information on connective properties	Usually low numbers of observations, highly dependent on injection site and volume
Cytoarchitectural approaches	High level of anatomical detail, information on chemical properties of cell populations	No information on connectivity, no functional data
Structural imaging	In vivo information	Low level of anatomical detail
Functional imaging	In vivo on distinct functions	Low level of anatomical detail
Clinical observations	Information on (dys)function	Low level of anatomical detail, usually low number of observations, not always normal brain function

## Topographical organization within the STN

### Tracing studies

Based on tracing studies using a variety of different tracers several groups have provided evidence for the existence of 0–4 anatomofunctional subdivisions within the STN (Alexander and Crutcher 1990; Parent and Hazrati 1995a, b; Joel and Weiner 1997; Keuken et al. 2012; Haynes and Haber 2013). A number of studies argue for the existence of three subdivisions with separate input and output pathways (Alexander and Crutcher 1990; Parent and Hazrati 1995a, b; Joel and Weiner 1997; Haber 2003; Hamani et al. 2004). Subthalamic neurons projecting to the putamen and to GPe are present in the dorsolateral part of the STN. Neurons projecting to the caudate nucleus and GPi and SNr were largely confined to the ventromedial part of the STN, although the projections to the striatum are not as well documented as those to the GP. Finally, neurons projecting to the ventral GP were confined to medial tip of the STN (Nauta and Cole 1978; Smith et al. 1990). Based on the anatomical connectivity, the dorsolateral part of the STN was defined as the motor part, the ventromedial part of the STN as the associative part, and the medial STN as the limbic subdivision of the STN (Fig. 1, Alexander and Crutcher 1990; Parent and Hazrati 1995a, b; Joel and Weiner 1997).

Tracing studies are technically challenging and caution should be used interpreting the data. Joel and Weiner (Joel and Weiner 1997) already pointed out that the body of evidence obtained from primates is interpreted in different ways by different researchers. The interpretation of these studies is complicated by inherent limitations of these experimental setups. Limitations include the comparison of results across different primate species, as well as modest numbers of observations. Intensity of the labeling may vary between injections, and injection sites are often small. This may sometimes lead to an underestimation of anatomical connectivity. Recently, Haynes and Haber (2013) published an extensive and well-executed tracing study in 43 macaques to map anatomical connections to different functional areas within the STN. 21 out of 48 injections were analyzed. This study confirmed the topographical organization of limbic, associative, and motor connections within the STN and provided additional anatomical detail (Haynes and Haber 2013). The authors report topographical organization within the STN based on the anatomical connections to different cortical regions. Overlap resulted from convergence of dense terminal fields from predominantly neighboring cortical regions, as well as from a wide spread of diffuse fibers resulting in overlap from more distant cortical areas. Haynes and Haber argue that projection fields of the STN may have been larger than reported due to the use of small injection sites.

The anatomical detail provided by Haynes and Haber is of importance for understanding side effects of DBS treatment in patients with Parkinson’s disease (Haynes and Haber 2013). It has been postulated that a subset of the deleterious side effects of DBS may be related to the location of the electric stimulator within the STN. Unintentional targeting of the limbic and associative parts of the STN has been hypothesized to cause limbic-behavioral side effects (Mallet et al. 2007).

### Conclusions tracing studies

**Fig. 2:**
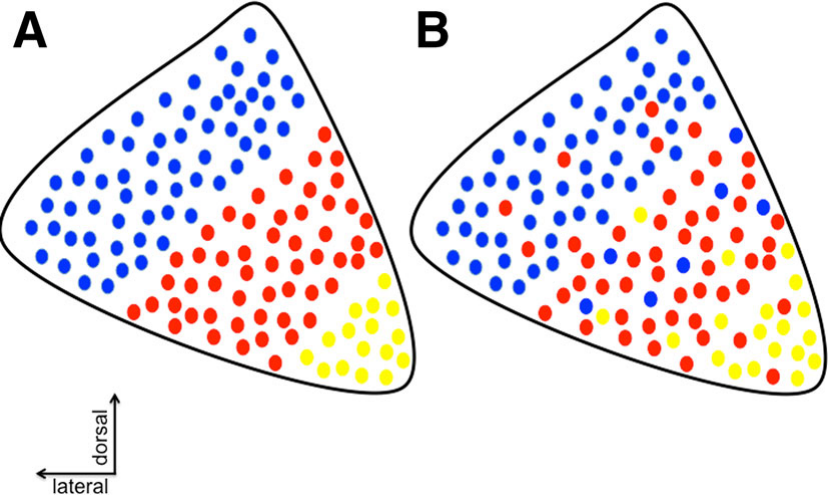
Topographical organization of the STN. **a** Schematic representation of anatomically distinct functional subdivisions/zones of the STN. **b** Alternative topographical organization of the STN without strict anatomically delineated subdivisions. Reproduced from (with permission) (Alkemade 2013)

Tracing studies in non-human primates provide evidence for topographical organization within the STN. In general, studies point toward topographical organization without clear borders in the STN (Haynes and Haber 2013; Alkemade 2013) (Fig. 2). Tracing studies are valuable, but also technically challenging. Caution should be used when interpreting these data, since the results are strongly affected by placement and volume of the tracer injection. In addition, conclusions are often drawn based on a limited number of observations (Alkemade 2013). We cannot exclude that a substantial overlap exists between the putative anatomofunctional subdivisions within the STN.

### Cytoarchitectural studies

It is feasible that different functions within the STN are regulated by different neurotransmitter systems. Strikingly little is known about the functional neuroanatomy of the human and non-human primate STN and results are often preliminary and sometimes even contradictory. A significant number of informative studies have been published on rodents. Since this review is focused on the human and non-human primate, we have not included studies on rodents.

#### Glutamate

The STN exerts a strong glutamatergic excitatory effect on the main basal ganglia output structures (Parent and Hazrati 1995a). The majority of subthalamic neurons were reported to show glutamate immunoreactivity in squirrel monkeys (Smith and Parent 1988). Glutamate acts via metabotropic glutamate receptors mGluR1a and 5 that are expressed in rhesus monkey STN (Kuwajima et al. 2004). Immunoreactivity of these two receptors shows significant overlap in STN and is concentrated in the synaptic area in close proximity of GABA-ergic synapses (Kuwajima et al. 2004). The data on glutamatergic output of the STN do not provide evidence for anatomically distinct subdivisions or zonations.

#### GABA

Although the main output of the STN is glutamatergic, a minority of STN cells express glutamate decarboxylase (GAD), which catalyzes the conversion of glutamate into GABA (Levesque and Parent 2005). The expression of GABA transporter (GAT) 1, which removes GABA from the synaptic cleft thereby terminating its’ action (Hirunsatit et al. 2009), was present in most STN neurons (Augood et al. 1999). These data are in line with other reports that the glutamatergic STN neurons receive a massive GABA-ergic innervation (Smith and Parent 1988), which fits with the expression of a number of GABA-A and -B receptor subunits as determined by in situ hybridization in monkeys (Kultas-Ilinsky et al. 1998; Charara et al. 2000). Like glutamatergic signaling, markers for GABA-ergic signaling do not provide evidence for anatomical subdivisions within the STN.

#### Dopamine

Tyrosine hydroxylase catalyzes the rate-limiting step in the synthesis of catecholamines and is therefore crucial for dopamine production. Although large numbers of tyrosine hydroxylase immunoreactive axons pass above and through the STN, these are presumed to mainly be axons passaging through in the direction of the neostriatum (Hedreen 1999). Occasional branches, which are morphologically indicative of axon terminals, innervated the STN (Hedreen 1999). The dopamine 1 receptor (D1R) was not expressed in human STN, whereas D2R showed weak expression (Hurd et al. 2001). Zonation was not investigated. A second study corroborated the absence of D1R from the STN; however, this study also reported a lack of D2R hybridization signal from the STN (Augood et al. 2000).

#### Serotonin

Serotonin strongly innervates monkey STN and shows clear topographical variation. The anterior half of the STN is more densely innervated as compared to the posterior half. Variation in expression was not present along any other of the brain axes (Parent et al. 2011). The human STN shows numerous serotonin transporter (SERT) immunoreactive varicosities, innervating STN neurons, which have not been further identified (Parent et al. 2011). The human STN contains a small number of thick and beaded SERT positive varicose fibers and a few thin and varicose fibers distributed according to a mediolateral decreasing gradient, whereas the isolated SERT positive axon varicosities appear uniformly distributed throughout the nucleus (Parent et al. 2011). Serotonin (5HT) itself cannot be detected in human postmortem tissue samples for technical reasons, but its’ distribution has been described in monkeys (Mori et al. 1985). Regional differences in 5HT fiber immunoreactivity were observed. Highest density was observed in the medial and ventral parts of the STN. In the rostral STN scattered tract fibers were observed. In the central and caudal parts, parallel fiber segments were oriented towards the lateral margin of the STN.

#### Enkephalin

Expression of receptors for endogenous opioids in the human STN was investigated using RNA blotting, which revealed the presence of several transcripts showing high expression in the STN (Raynor et al. 1995). mRNA expression of the prepro-Enkephalin B gene was observed in the STN in monkeys. An increased expression was present during levodopa treatment in a model for Parkinson’s disease in monkeys. However, STN subdivisions were not investigated (Aubert et al. 2007).

#### Calcium-binding proteins

Calcium-binding proteins are used to differentiate between distinct populations of interneurons in the basal ganglia (Parent et al. 1996). The distribution of calcium-binding proteins in the human STN shows clear zonation. Calretinin-immunoreactive neurons are particularly present in the ventromedial part of the human STN (Parent et al. 1996; Augood et al. 1999), parvalbumin is present in the dorsolateral part of the STN, whereas calbindin does not appear to be expressed in human STN (Augood et al. 1999). Despite the difference in zonal expression of calretinin and parvalbumin, there was a significant overlap in their immunoreactivity (Augood et al. 1999). These data support a topographical organization without clear anatomical boundaries within the STN.

### Conclusions cytoarchitectural studies

Evidence from postmortem studies supports a topographical organization within the STN based on the distribution of several markers found within the nucleus. These findings are summarized in Table 2. Markers showing an inhomogeneous distribution over the STN, however, do not provide evidence for clear topographically delineated subdivisions within the STN. Translation of cytoarchitectural studies into function would rely on speculation, but the complexity of the cytoarchitectural staining patterns may suggest the existence of a number of overlapping neuronal populations within the STN. It is possible that overlapping neuronal populations with distinct neurochemical characteristics act in concert to modulate STN output.

**Table 2:** Summary of histological observations

Protein/mRNA	Function	Human/monkey	Zonation/subdivision	References
mGlur1a	G-protein-coupled receptors for glutamate	M	None reported	Kuwajima et al. (2004)
mGlur5	G-protein-coupled receptors for glutamate	M	None reported	Kuwajima et al. (2004)
GAD	Glutamate decarboxylase, catalyzes the conversion of glutamate to GABA	H	None reported	Levesque and Parent (2005)
GAT1	GABA transporter 1	H	None reported	Augood et al. (1999)
GABA-A receptor	Ligated ion channels	M	None reported	Kultas-Ilinsky et al. (1998)
GABA-B receptor	G-protein-coupled receptors	M	None reported	Charara et al. (2000)
TH	Tyrosine hydroxylase, rate-limiting enzyme in catecholamine production	H	None reported	Hedreen (1999)
DR2	Dopamine Receptor	H	None reported, expression in STN is controversial	Hurd et al. (2001); Augood et al. (2000)
SERT	High affinity serotonin reuptake transporter	H	More staining in anterior STN as compared to posterior	Parent et al. (2011)
5HT	Serotonin	M	Highest density in medial and ventral part of the STN	Mori et al. (1985)
ppEnkB	prepro-Enkephalin B encodes Endogenous opioid	M	None reported	Aubert et al. (2007)
Parvalbumin	Calcium-binding protein	H	Clear zonation, higher dorsolateral expression	Parent et al. (1996); Augood et al. (1999)
Calretinin	Calcium-binding protein	H	Clear zonation, higher ventromedial expression	Parent et al. (1996); Augood et al. (1999)

## In vivo imaging

### Structural MRI

**Fig. 3:**
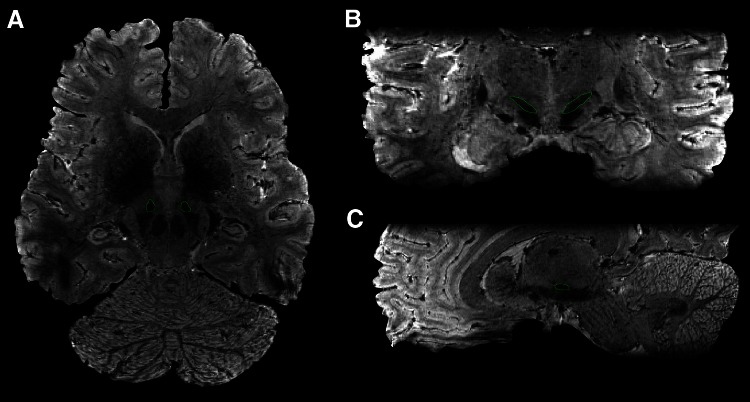
Illustration of STN visualization using T2*-weighted 0.5 mm^3^ isotropic 7T MRI. **a** Transverse view; **b** coronal view; **c** sagittal view

MRI techniques and equipment have rapidly developed over the years. 3 Tesla imaging techniques have been successfully applied in basic studies on the STN, as well as surgical targeting of the STN (e.g., Lambert et al. 2012; Polanski et al. 2015). Using state-of-the-art ultrahigh-resolution 7 Tesla imaging techniques it is now possible to visualize the border between the STN and SN in vivo (Cho et al. 2010; Turner 2012; Keuken et al. 2012) (Fig. 3). The improved contrast is promising for the improvement of anatomy guided DBS (Abosch et al. 2010). Abosch et al. (2010) defined three criteria along which optimal MRI for delineation of the STN could be developed. Ideally, imaging for clinical application including stereotactic guided DBS surgery needs to provide 3 qualities: the highest possible signal to noise ratio, the highest possible image resolution with adequate contrast, and the minimum possible image distortion (Abosch et al. 2010). We have attempted to use iron to identify local differences within the STN (de Hollander et al. 2014). Although there were clear gradients of iron concentrations within the nucleus, no anatomical subdivisions of the STN could be distinguished. Our efforts represent a first step in performing in vivo quantifications in the STN. Interestingly, a recent study utilized probabilistic tractography to investigate STN subdivisions using cluster analyses (Lambert et al. 2012). These studies support a somatotopic organization within the nucleus. The authors suggest that it may be the case that unique limbic and motor STN zones exist, and that the associative zone presents an overlapping, somatotopically arranged transition between the two.

## Functional imaging

### Motor function

Despite the current interest, still very little is known about the STN’s normal function in relation to movement. Movement is closely correlated with STN and pallidal activations (Toxopeus et al. 2012). The impact of STN dysfunction on movement is illustrated by the striking effects of STN lesions, which cause involuntary movements in contralateral extremities (Crossman et al. 1984). In addition, disruption of STN function causes impulsive responses (Frank et al. 2007). A very recent study has investigated brain activation in healthy participants in relation to different aspects of movement. Ballistic movement initiation, stepwise interrupted movement, step track, and continuous circle movements were compared (Toxopeus et al. 2012). Movement initiation was related to activations in a number of areas including contralateral SN, caudate head, bilateral putamen, and the posterior thalamus. STN activation was related to movement inhibition, as was activation in the ipsilateral pallidum, striatum, and dorsolateral prefrontal cortex. Activations related to gradually modulated movement and visuomotor control were located in the bilateral pallidum, posterior dorsal putamen, bilateral cerebellum, and primary motor cortex among other areas (Toxopeus et al. 2012). These data suggest that STN involvement in motor control is restricted to effects on motor inhibition.

### Associative function

The role of the STN in motor inhibition has been studied in more detail. An increase in BOLD signal in the subthalamic region has been observed in stop signal tasks (Aron and Poldrack 2006; Li et al. 2008). A white matter network including the STN, pre-supplementary motor area, and inferior frontal cortex was uncovered in the right hemisphere using diffusion-weighted MRI (DWI) tractography (Aron et al. 2007; Boehler et al. 2010; Chikazoe 2010). In addition, a hyperdirect pathway from cortex via STN has been implicated (Aron and Poldrack 2006; Aron et al. 2007; Isoda and Hikosaka 2008). In this network, the STN is considered to act as a relay station for action inhibition, and these effects may be mediated via alterations in gamma oscillations in the cortico-subthalamic connection (Aron and Poldrack 2006; Frank 2006; Eagle et al. 2008; Alegre et al. 2013).

### Limbic function

Most reports on the limbic functions of the STN are related to observations in pathology. Only few studies have investigated emotional processing. Bartels and Zeki (Bartels and Zeki 2004) found activation in the STN area in relation to maternal love. In addition, Karama et al. (Karama et al. 2011) showed that emotional movies induced STN activation. These studies unfortunately did not use high-resolution scanning so that a detailed anatomical delineation of the STN was not possible.

## Conclusions in vivo imaging

In line with postmortem data, in vivo imaging studies support zonation within the STN. Unfortunately, especially older studies do not provide sufficient anatomical detail to allow precise delineation of the STN. In these studies, often an STN region containing the nucleus but also some surrounding areas may have been included in the analyses.

## Clinical observations

The role of the STN in motor function is evident from STN lesions that produce violent, involuntary, flinging movements known as ballism. Ballism is generally confined to the side of the body contralateral to the lesion (Parent and Hazrati 1995a, b). A case report described hemiballism, persistent hypersexuality, as well as memory and executive dysfunction in a patient after a subthalamic infarction (Absher et al. 2000). Several studies have aimed to investigate involvement of the STN in PD pathology. A decreased STN BOLD signal was observed in PD patients who had freezing of gait as compared to PD patients who did not (Shine et al. 2013). Dysinhibition of the STN by loss of dopaminergic input is thought to contribute to the motor symptoms of Parkinson’s disease. DBS improves these motor symptoms, but also affects heart rate, mood, motivation, and sexual behavior (Kumar et al. 1998; Limousin et al. 1998; Krack et al. 2000). Common side effects include hypophonia, apathy, euphoria/hypomania, stroke, and worsening of depressive illness (Thobois et al. 2002; Wertheimer et al. 2014; Witt et al. 2012) (Doshi et al. 2002). It should be noted that side effects described in patients treated with DBS may in some cases be related to the cessation of L-DOPA treatment (Connolly and Lang 2014). Patients have also been described to exhibit difficulties in their relations with themselves, their spouses, their families, and their social and professional environment (Schupbach et al. 2006). Transient mania, pseudobulbar crying, and anxiety have also been reported (Visser-Vandewalle et al. 2005; Chang et al. 2012). However, in studies only including patients without prior cognitive and psychiatric disorders, STN stimulation improved mood, anxiety, and quality of life without causing permanent psychiatric disorders or modifying personality, or modifying social functioning (Houeto et al. 2006). DBS of the STN induces abnormal impulsivity in some PD patients, as illustrated by poor inhibition in conflict-associated decision processes (Jahanshahi et al. 2000; Frank et al. 2007), or when withholding movements (Ray et al. 2012).

Several studies have attempted to correlate the motoric as well as the unwanted side effect of DBS to the anatomical location of the electrical stimulator. Clinical observations in two patients with hypomanic side effects have shown that the hypomanic state was caused by stimulation via the DBS contact located in the anteromedial STN. The anteromedial contact, as well as a contact more dorsal improved motor symptoms. Contacts at the boundaries of the STN affected neither behavior, nor motor performance (Mallet et al. 2007). The specific properties of the DBS electrodes (placement, current strength) are crucial to the exerted clinical effect, especially since DBS leads are relatively large (usually 4 contacts of 1.5 mm separated by 0.5 or 1.5 mm), and currents inherently spread through the tissue to a certain extent.

## Conclusions clinical observations

A number of medical conditions confirm the role of the STN in movement, as well as associative, and limbic processes. Some evidence for STN zonation or subdivisions is present. These studies are complex due to the number of different functions in which the STN is involved. Additionally, studies are further complicated by factors such as age, possible concomitant illness, and a lack of control groups for comparison. Finally, it is important to realize that confirmation of probe placement is not trivial.

## Electrophysiological recordings in DBS patients

In addition to the therapeutic benefits of DBS surgery, recordings during the implantation procedure have greatly improved our understanding of the electrophysiological properties of the human STN. It is important to note that for obvious reasons such recordings can only be performed in patients with a medical need for DBS. It therefore remains unclear to which extent the observations can be translated to normal STN function. Electrophysiological recordings in PD patients support the presence of a motor subdivision in the dorsolateral part of the STN (Rodriguez-Oroz et al. 2001). This was assessed by measuring modulation of the neuronal discharge in relation to passive and active movements of the contralateral limbs (Rodriguez-Oroz et al. 2001; Abosch et al. 2002). Comparison of electrophysiological properties of obsessive–compulsive disorder (OCD) and PD STN’s revealed differences in oscillation characteristics of STN neurons. STN firing rates in OCD were lower than in PD. In line with this, OCD patients showed longer interspike intervals. Interestingly, the interspike interval was longer in the putative motor area as compared to the limbic/associative subdivision of the STN (Welter et al. 2011). These data suggest that different parts of the STN are involved in the pathogenesis of OCD and PD.

Coherence has been widely used to quantify similarity between neuronal oscillations and is commonly interpreted as interaction or communication between brain areas (Fries 2005; Schnitzler and Gross 2005). Topographical differences of tremor-associated coherence suggest that the subthalamic area in patients with PD is further organized in distinct segregated ‘tremor clusters’ that are specific for rest and postural tremor activity (Reck et al. 2010). By combining magnetoencephalography (MEG) and local field potentials (LFP), frequency-dependent interactions between STN and cortex have been mapped. These studies have revealed distinct couplings between STN and cortex in PD patients: One with the motor cortex in the beta frequency band and one with temporal areas in the alpha frequency band (Hirschmann et al. 2011). Several studies in PD patients have shown an increased beta-band activity in this area, especially in the dorsal region of the STN (Chen et al. 2006; Weinberger et al. 2009). Further studies have revealed theta coherence in mesial and lateral subthalamic area, alpha and lower beta coherence in the mesial and ipsilateral motor areas, in addition to upper beta coherence the midline cortex. LFPs in the subthalamic areas led electroencephalography (EEG) in the theta band. In contrast, EEG led the depth LFP in the lower and upper beta bands. LFP activity in the alpha band could either lead or lag EEG. Thus, there are several functional sub-loops between the subthalamic area and cerebral cortical motor regions, distinguished by their frequency, cortical topography, and temporal relationships (Fogelson et al. 2006). These data support the presence of subdivisions or zonation within the STN. Unfortunately, the resolution was too low to firmly establish if recordings were made from the STN, or as stated by the authors in the subthalamic area.

Further studies in PD patients revealed that pleasant, unpleasant, and neutral stimuli evoked an event-related potential (ERP). The magnitude of the effects was maximal in the ventral part of the STN and dependent on dopamine medication (Buot et al. 2012).

## Conclusions electrophysiological recordings in DBS patients

DBS stimulation has profound effects on STN function, although stimulation of adjacent brain areas cannot be excluded (Fontaine et al. 2004; Mallet et al. 2008).

## Concluding remarks

The need for a better understanding of the STN’s functional neuroanatomy is evident, both for increasing insight in the pathogenesis of movement disorders as well as optimization of DBS for improvement of motor symptoms and prevention of unwanted side effects. In all of the studies describing anatomofunctional subdivisions or zones within the STN we have reviewed, the respective authors carefully discuss the existence of topographical overlap between the subdivisions. The magnitude of the overlap is of clinical relevance. With the development of new high-resolution MRI techniques in combination with in vivo electrophysiological measures during stereotactic surgery, and technical advancements in the available electrical stimulators, DBS will potentially become even more valuable in the future. It is feasible that neurosurgeons will be able to selectively target the specific parts of the STN. However, we feel that at present the evidence supporting the existence of subdivisions of the STN without information on the degree of overlap between these subdivisions is insufficient to provide surgeons with specific targets within the STN. We have reviewed support for STN subdivisions from different research disciplines within the field of neuroscience. A topographical organization within the STN may be present; however, it remains unclear to what extent functional and anatomical subdivisions/zones overlap. This review and recent publications by us and others (Alkemade and Forstmann 2014; Lambert et al. 2015) indicate that the discussion on the tripartite subdivision hypothesis of the STN is still ongoing. This discussion may inspire new anatomical studies as well as studies developing computational models aimed to answer questions on parallel vs. convergent/divergent signaling.
